# Opportunities and challenges in early diagnosis of rheumatoid arthritis in general practice

**DOI:** 10.3399/bjgp23X732321

**Published:** 2023-03-31

**Authors:** Heidi J Siddle, Stephen H Bradley, Anna M Anderson, Kulveer Mankia, Paul Emery, Suzanne H Richards

**Affiliations:** Health Education England (HEE)/National Institute for Health and Care Research (NIHR) Senior Clinical Lecturer, Associate Professor, and Consultant Podiatrist, Leeds Institute of Rheumatic and Musculoskeletal Medicine, University of Leeds, Leeds.; Leeds Institute of Health Sciences, University of Leeds, Leeds; Leeds Institute of Health Sciences, University of Leeds, Leeds.; Leeds Institute of Rheumatic and Musculoskeletal Medicine, University of Leeds; Consultant Rheumatologist, NIHR Leeds Biomedical Research Centre, Leeds Teaching Hospitals NHS Trust, Leeds.; Leeds Institute of Rheumatic and Musculoskeletal Medicine, University of Leeds; Consultant Rheumatologist, NIHR Leeds Biomedical Research Centre, Leeds Teaching Hospitals NHS Trust, Leeds.; Professor of Health Services Research, Leeds Institute of Health Sciences, University of Leeds, Leeds.

## INTRODUCTION

Prompt diagnosis of rheumatoid arthritis (RA), the most common form of inflammatory arthritis, is crucial to optimise long-term patient outcomes through prevention of joint damage and disability. However, early disease can be challenging to identify in primary care, especially given that RA makes up a small proportion of the musculoskeletal conditions that account for one in seven GP appointments.^1^ Patients consult with GPs a mean of four times before being referred to rheumatology services.^2^ The non-specific nature of symptoms at the earliest stages of RA is a barrier to GPs identifying patients with newly presenting RA.

## IDENTIFYING EARLY DISEASE

The rationale for identifying early disease is to initiate treatment using disease-modifying therapies (including biologics) in the reversible stage of the disease, referred to as the RA ‘therapeutic window of opportunity’ in the 3 months following the onset of clinical synovitis.^3^ This can significantly improve clinical outcomes and health-related quality of life, with earlier disease control reducing work-related disability.

However, the discovery that circulating autoantibodies, including anticitrullinated protein antibodies (ACPA), precede the clinical onset of disease provides an opportunity to identify people with musculoskeletal symptoms who are at risk of developing RA.^4^ ACPA can be identified through an anti-cyclic citrullinated peptide (anti-CCP) test. A high positive anti-CCP result is more specific for joint pathology than rheumatoid factor, and is strongly associated with the development of RA (Table 1).^4^^–^^6^

**Table 1.** Diagnostic test properties of the anti-CCP test

**NICE guidance NG100 on anti-CCP testing for RA in adults**	
*‘1.1.3 Consider measuring anti-CCP antibodies in adults with suspected RA if they are negative for rheumatoid factor’*	
**Anti-CCP test serological categories^6^**	
Negative	≤ULN
Low-level positive	>ULN ≤three times ULN
High-level positive	>three times ULN
**Anti-CCP2 test for predicting development of RA among people presenting to primary care with new-onset, non-specific MSK symptoms without synovitis, % (*n*/*N*)^4^^,^^a^**	
Percentage of anti-CCP-positive individuals	2.8 (57/2028)
Percentage of anti-CCP-positive individuals who developed RA	42.1 (24/57)
Relative risk for developing RA within 12 months in the anti-CCP-positive group (95% CI)	66.8 (32.2 to 138.4), *P*<0.001
Sensitivity (95% CI)	64.9 (48.8 to 78.2)
Specificity (95% CI)	97.9 (97.1 to 98.5)
PPV (95% CI)	42.1 (30.2 to 55.0)
NPV (95% CI)	99.2 (98.6 to 99.5)
**Anti-CCP2 test categorised as high- or low-level positive, combined with clinical symptoms, for predicting development of IA among people presenting to primary care with new-onset, non-specific MSK symptoms without synovitis, % (*n*/*N*)^5^^,^^b^**	
Percentage of anti-CCP high-level positive individuals who developed IA	62.2 (61/98)
Percentage of anti-CCP low-level positive individuals who developed IA	13.2 (7/53)
PPV, high-level positive, presence of hand or foot pain (95% CI)	69.1 (63.9 to 73.9)
NPV, low-level positive, absence of hand and foot pain (95% CI)	95.8 (78.6 to 99.3)

a *Data from 2028 individuals in the Leeds anti-CCP cohort. Follow-up data on development of RA was available for 1614 individuals. The median (interquartile range) length of follow-up for the anti-CCP-positive and anti-CCP-negative individuals was 11.5 (1.5–28.2) months and 13.8 (12.5–21.5) months, respectively.*

b *Data from 151 anti-CCP positive individuals in the Leeds anti-CCP cohort — 63/68 (92.6%) of the anti-CCP-positive individuals who developed IA met the criteria for RA. The mean (standard deviation) length of follow-up for the high-level and low-level-positive individuals was 91 (122.1) weeks and 133 (117.2) weeks, respectively. Anti-CCP = anti-cyclic citrullinated peptide. Anti-CCP2 test = second-generation anti-cyclic citrullinated peptide test. IA = inflammatory arthritis. MSK = musculoskeletal. NICE = National Institute for Health and Care Excellence. NPV = negative predictive value. PPV = positive predictive value. RA = rheumatoid arthritis. ULN = upper limit of normal*.

The international rheumatology community has adopted the term ‘pre-RA’ to retrospectively describe a phase that an individual has progressed through once it is known that they have developed RA.^7^ It is during this period that patients may present in primary care with non-specific musculoskeletal symptoms. Secondary care models in autoantibody-positive patients have evolved to predict the early development of RA before synovitis is clinically apparent. However, the applicability of these models to primary care is unknown: non-specific musculoskeletal symptoms are common in the community, and the presence of RA-related autoantibodies (ACPA) may have important differences in natural history and prognosis when identified in those with non-specific musculoskeletal symptoms compared with disease that presents with clinical synovitis.

New research from the Leeds anti-CCP cohort, analysing 6780 patients from 312 general practices throughout England, demonstrated that individuals with high anti-CCP levels and joint pain in their hands/feet (without synovitis) have an increased likelihood of developing RA, compared with those with low anti-CCP levels (Table 1).^5^ Targeted anti-CCP testing in general practice could identify people at high risk of developing RA, enabling referral to rheumatology services prior to the development of synovitis to facilitate monitoring, diagnosis, and rapid initiation of treatment.^5^

## ANTI-CCP TESTING IN PRIMARY CARE FOR NON-SPECIFIC MUSCULOSKELETAL SYMPTOMS

Identifying pre-RA is a ‘needle in the haystack’ in primary care due to the myriad of musculoskeletal presentations. Changing the diagnostic paradigm of RA to detection prior to the onset of classical clinical synovitis requires robust evidence regarding the appropriate selection of patients ‘at risk’ of RA in primary care, and that targeted anti-CCP testing results in overall benefit, minimises harms, and is cost-effective.

Research is underway to develop criteria to identify people presenting to primary care with new-onset musculoskeletal symptoms who are likely to be anti-CCP positive. Economic modelling is also exploring the cost-effectiveness of such testing, considering the workload implications within general practice and rheumatology services, the resources needed to support interpretation of test results, and pathology costs of widespread anti-CCP testing.

## OPPORTUNITIES AND CHALLENGES OF IDENTIFYING PRE-RA

Even if primary care prediction models perform adequately, evidence is required regarding the clinical- and cost-effectiveness and safety of ‘pre-RA’ intervention. The benefits of treating pre-RA may include reducing the risk of clinical outcomes associated with comorbidities, such as cardiovascular disease-related mortality in RA (relative risk 1.48 [95% confidence interval = 1.36 to 1.62]).^8^ New evidence is emerging to support an earlier therapeutic window, with disease-modifying treatments to halt the biological processes and prevent the onset of RA being tested within clinical trials.^9^ There are, however, substantial adverse effects of disease-modifying therapies, and it should not be assumed that evidence on the balance of benefits and harms found for patients with RA diagnosed following presentation with typical symptoms is generalisable to the pre-RA population.

’Pre-RA’ must, therefore, be recognised as a different entity from RA. Potential harms of a strategy that will label patients as having pre-RA must be considered, such as increased anxiety, reluctance to undertake usual levels of activity due to perceived disability, or wider social implications such as increased costs of insurance policies or restriction of occupational opportunities. The scale of such harms will depend on the extent of overdiagnosis that can be expected, that is, the proportion of individuals labelled at risk who would not have gone on to develop RA (Table 1). While we understand the clinical risk factors for RA development in the at-risk population,^10^ there is still potential for a high rate of false positive anti-CCP tests (Table 1) and it is not yet understood how frequently we should monitor these clinical risk factors. The optimal primary and secondary care service models to monitor and support patients, and the associated workload and resource implications, also require further research.

Potentially modifiable lifestyle risk factors such as raised body mass index and smoking are strongly associated with the development of RA.^10^ Our recent systematic review highlighted that individuals at risk of RA have a need for more knowledge about RA and their potentially modifiable risk factors, which in turn could support their engagement with preventive interventions.^11^ However, as yet there is no clear indication that modifying these lifestyle risk factors will prevent or delay the onset of disease.

Further evidence is also needed to determine if disease-modifying therapies can prevent or delay the onset of RA. Accordingly, our team are currently recruiting those with musculoskeletal symptoms who have tested positive for anti-CCP antibodies and who are at moderate or high risk of developing RA using a risk-stratification prediction model (antibody concentration more than three times the upper limit of normal plus hands/feet tenderness, and/or ≥30 minutes early-morning stiffness) to participate in a therapeutic intervention study (48-week 2 mg daily oral dose of baricitinib) to determine if it reduces the incidence of RA.^9^

## CONCLUSIONS

Non-specific musculoskeletal symptoms constitute a large proportion of all consultations in primary care. Testing some of these patients using anti-CCP may provide a means to identify those at risk of RA and potentially delay or prevent its onset. Before these potential benefits can be adequately realised, further research is required to evaluate and mitigate countervailing harms and costs of such a strategy, and to understand how widespread testing can be integrated into routine primary care in a way that is acceptable to GPs and patients.
